# Editorial Note: Global Gene Expression Analysis of Canine Osteosarcoma Stem Cells Reveals a Novel Role for COX-2 in Tumour Initiation

**DOI:** 10.1371/journal.pone.0308114

**Published:** 2024-08-15

**Authors:** The PLOS ONE Editors

Following the publication of this article [1], concerns were raised that the Fig 1G Fibronectin results of [1] appears similar to the Fig 1D Fibronectin result of [2] despite being used to represent results from different cell lines.

The corresponding author stated that the Fig 1G Fibronectin results were correctly reported in the *PLOS ONE* article [1] but were inadvertently duplicated in [2]. They stated that all cell lines were blotted together, and that the incorrect lanes were selected when preparing the Fibronectin results presented in [2].

The available original data underlying Fig 1 [1] are provided in S1–S5 Files. The original blot image underlying the Fig 1G β-catenin panel is no longer available. The authors did not clarify the availability of the remaining data.

The *PLOS ONE* Editors issue this Editorial note to notify readers of the above concern, which PLOS considers resolved, and to share the available original data for Fig 1 that have been provided by the corresponding author.

## Supporting information

S1 File Original data underlying Fig 1A and 1B. (PPTX)

S2 File Original data underlying Fig 1D. (PPTX)

S3 File Individual level data underlying Fig 1E. (XLS)

S4 File Original data underlying Fig 1F. (PPTX)

S5 File Original data underlying Fig 1G. (PPTX)
