# Supplementary figures and images for: Editorial Note: Global Gene Expression Analysis of Canine Osteosarcoma Stem Cells Reveals a Novel Role for COX-2 in Tumour Initiation
Source: PLoS One. 2024 Aug 15;19(8):e0308114. doi: 10.1371/journal.pone.0308114 (PMC11326644; doi:10.1371/journal.pone.0308114)

## Slide 1
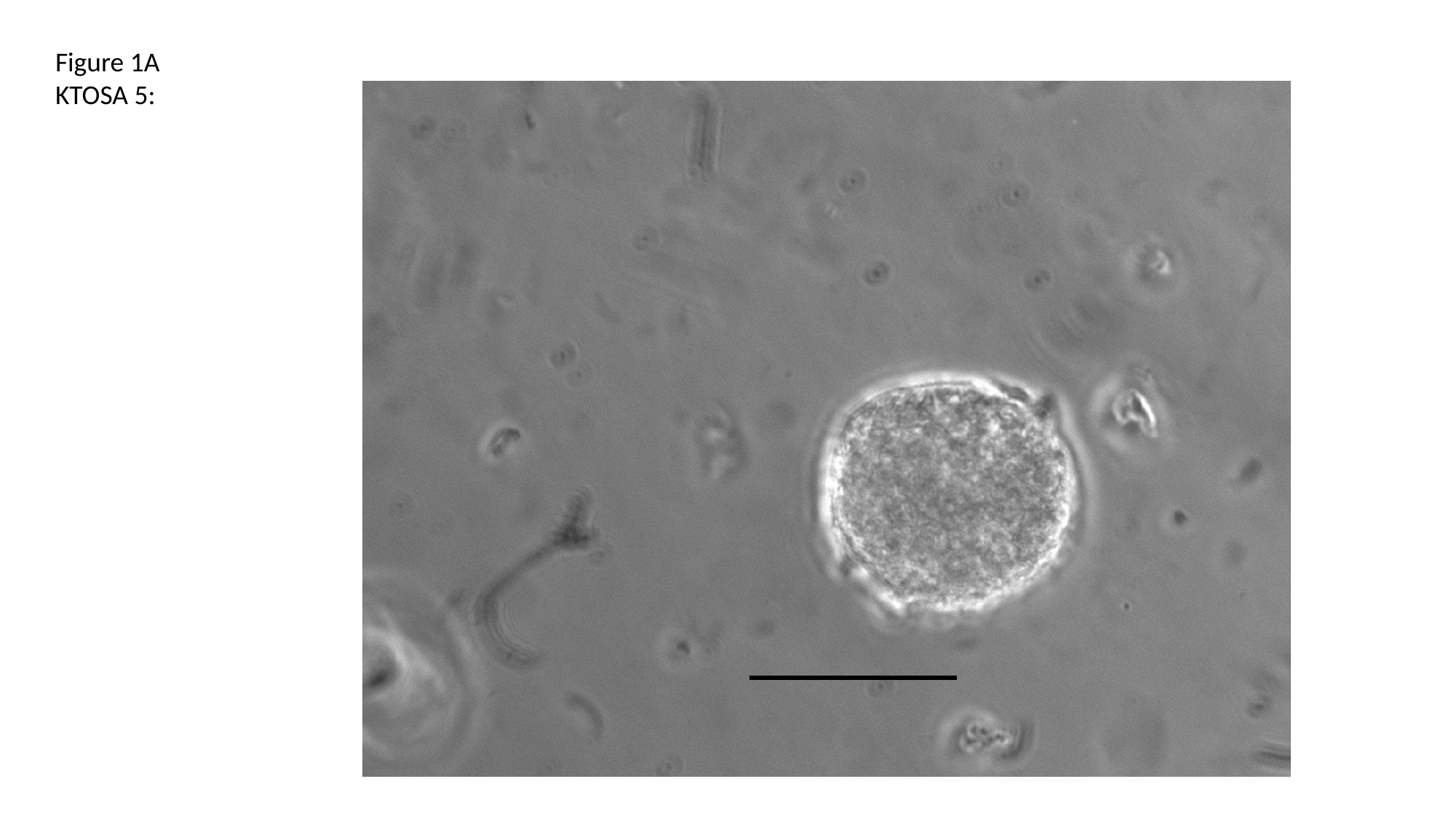

Figure 1A
KTOSA 5:

## Slide 2
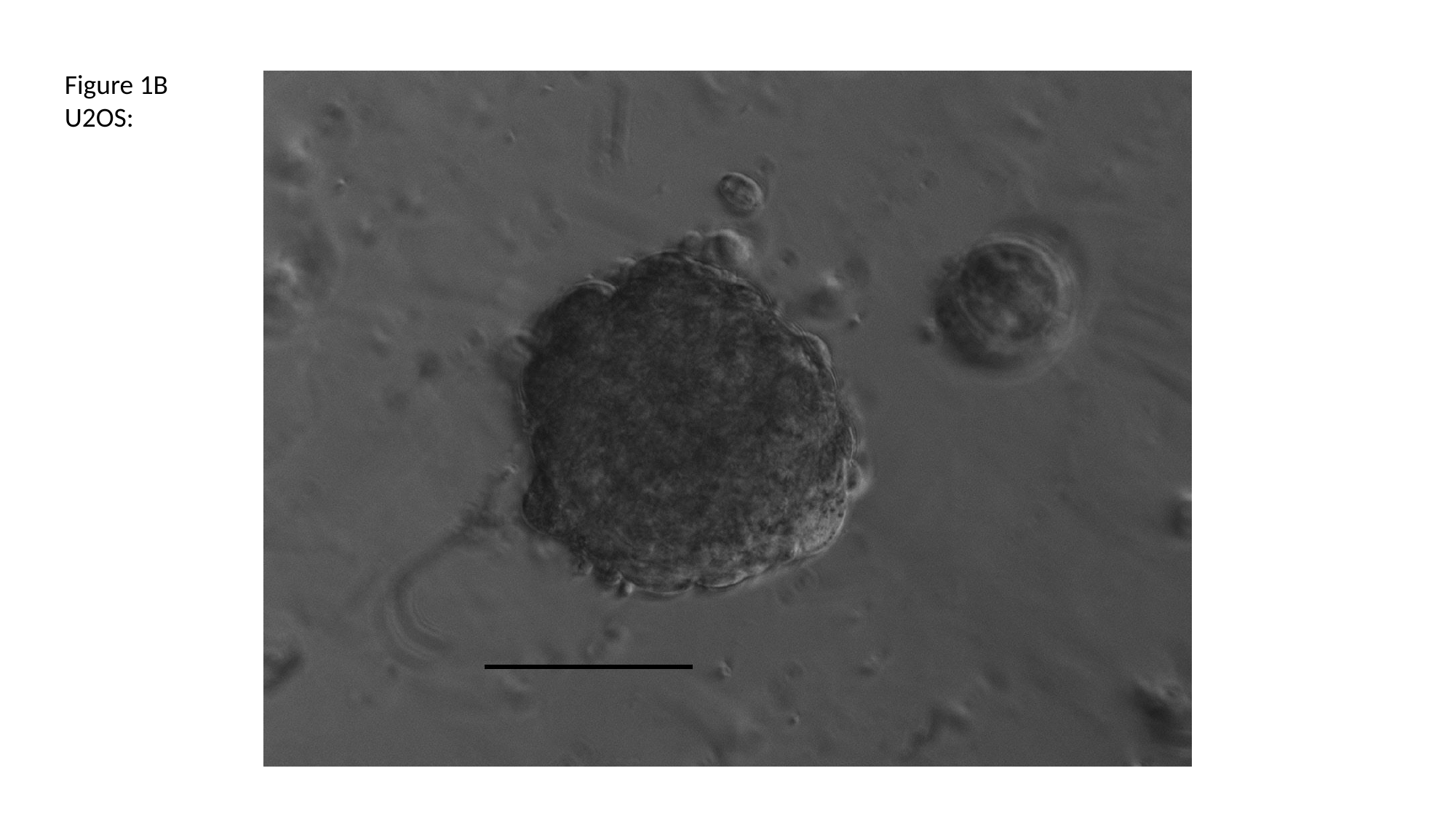

Figure 1B
U2OS:

Supplement: S1 File — (PPTX) [file pone.0308114.s001.pptx]

## Slide 1
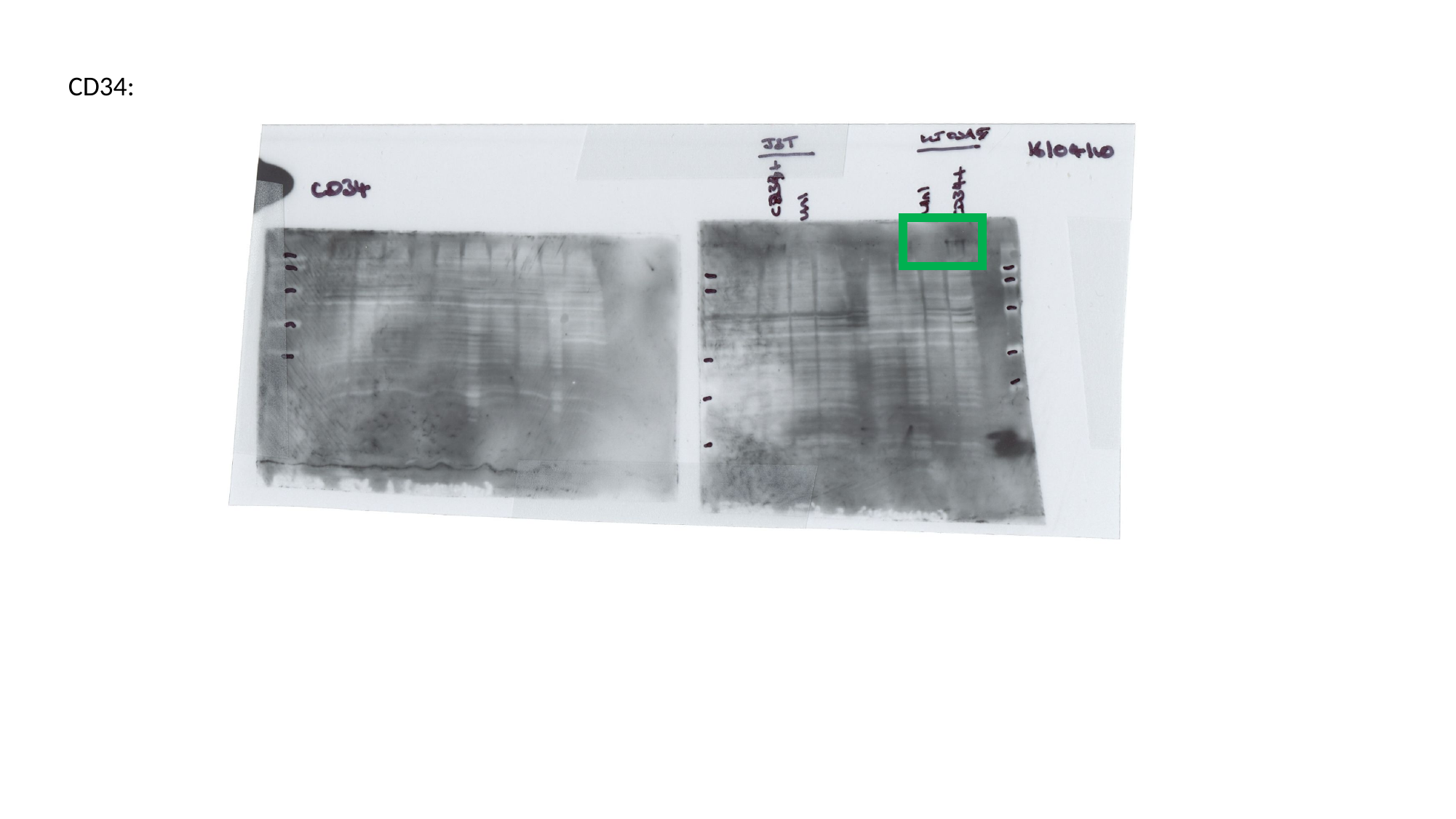

CD34:

## Slide 2
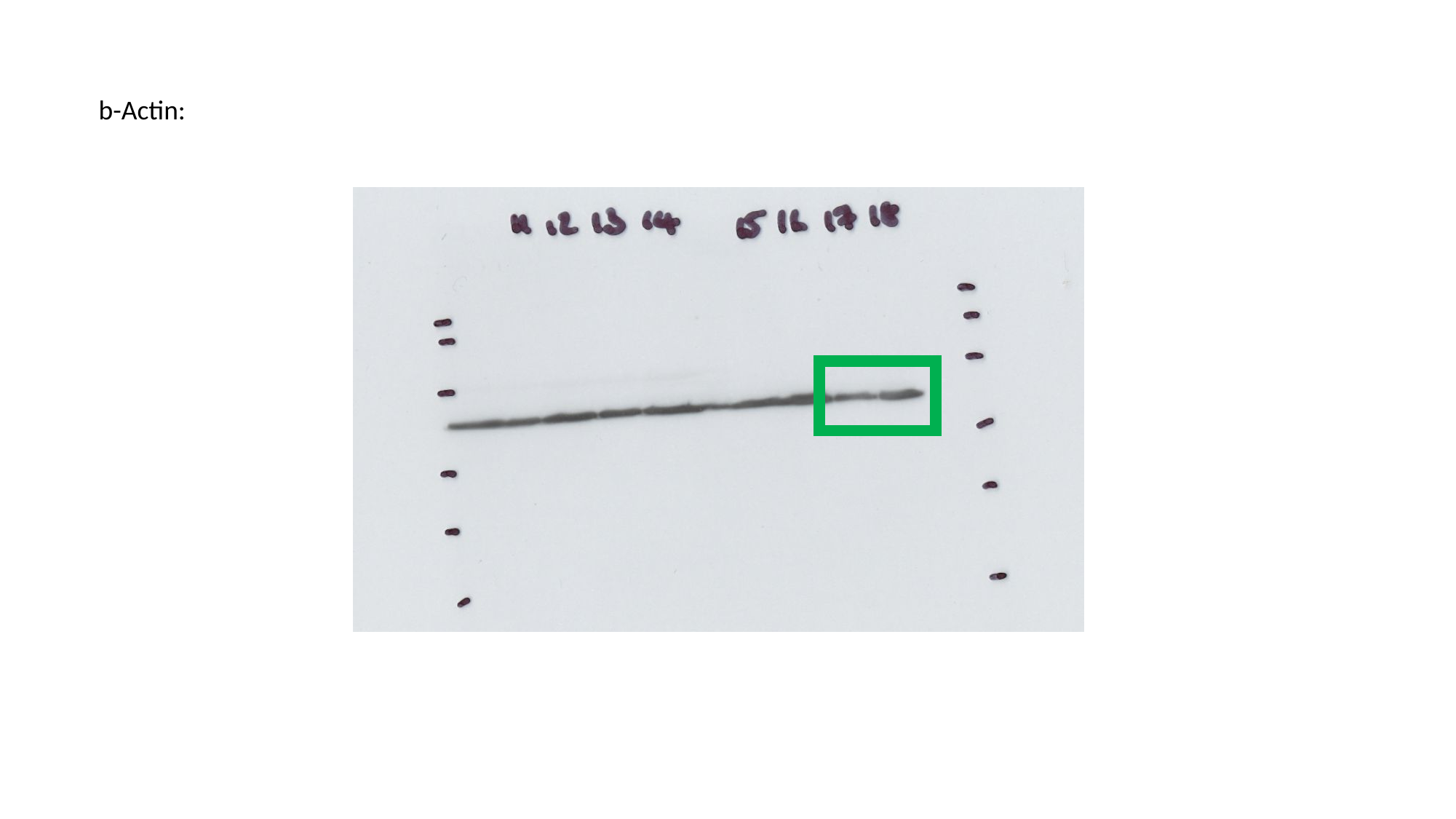

b-Actin:

Supplement: S2 File — (PPTX) [file pone.0308114.s002.pptx]

## Slide 1
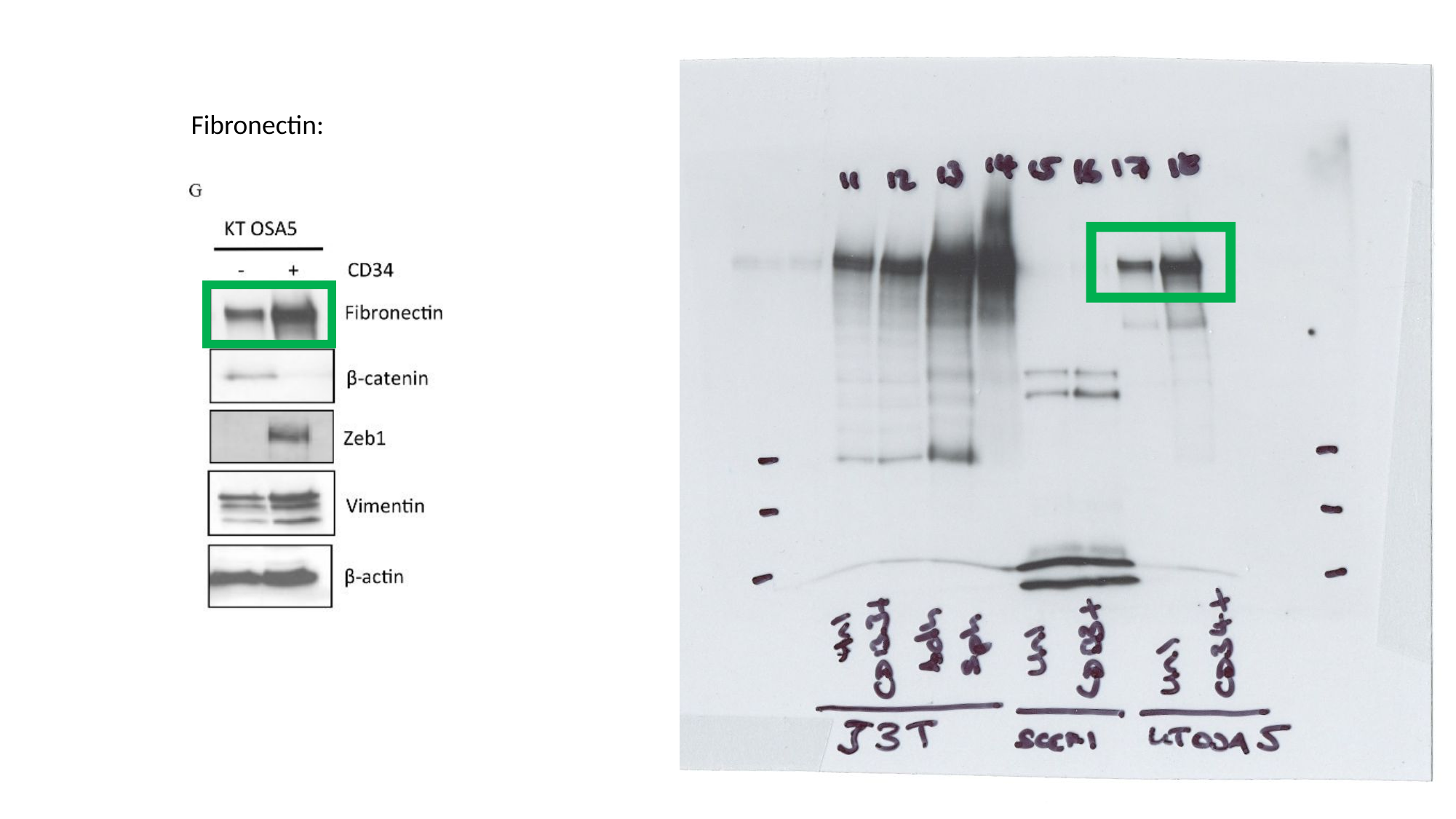

Fibronectin:

## Slide 2
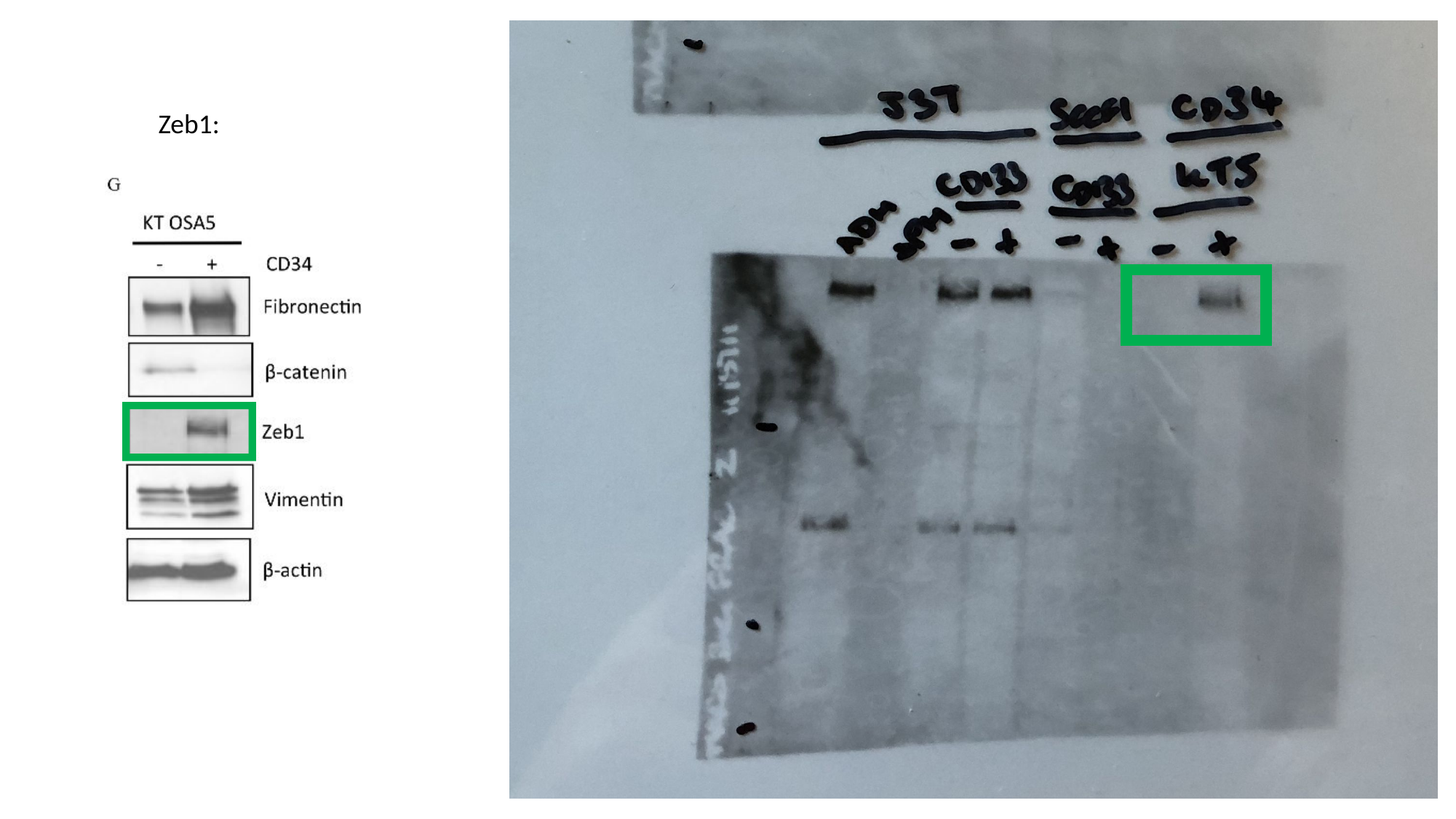

Zeb1:

## Slide 3
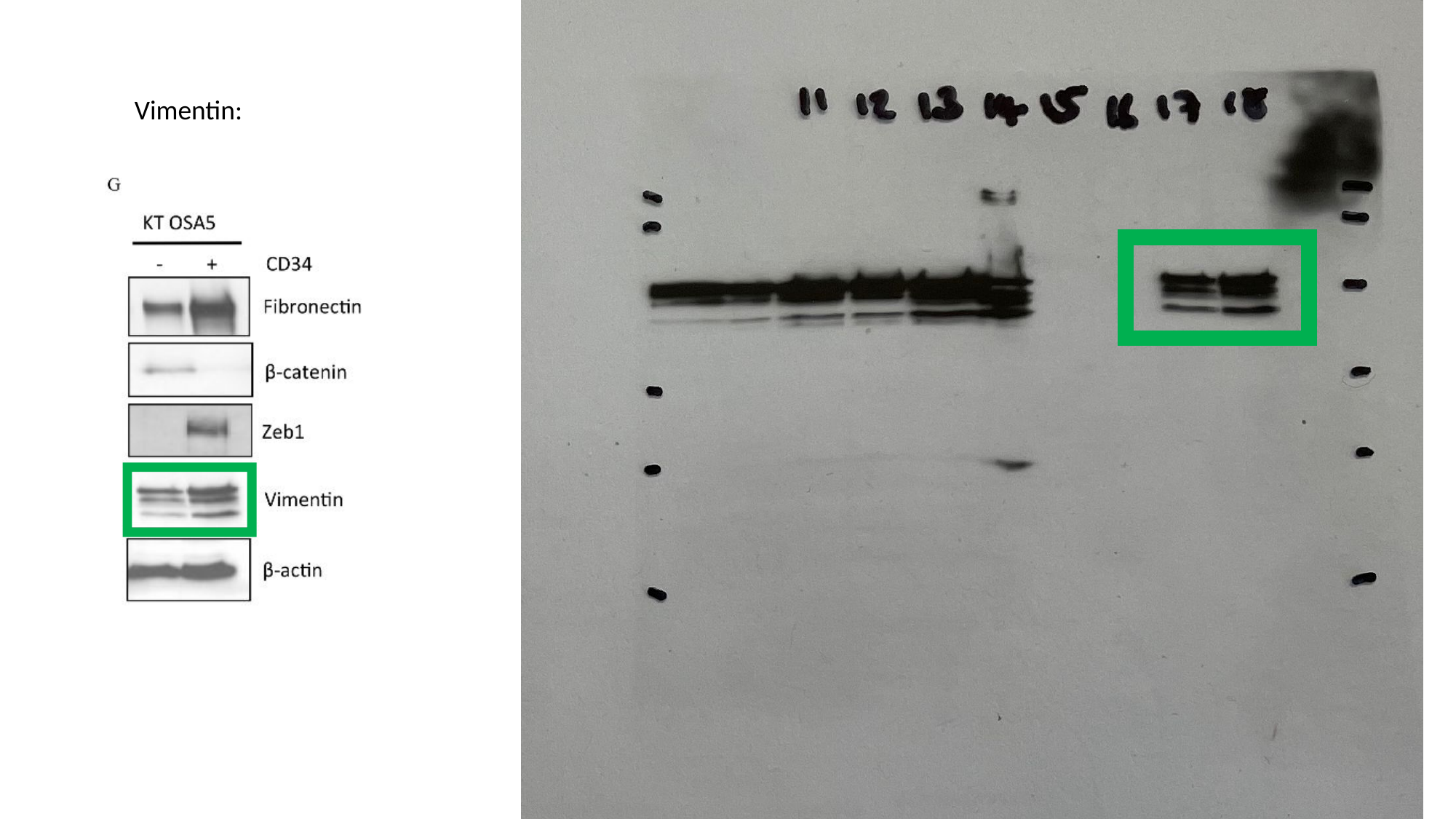

Vimentin:

## Slide 4
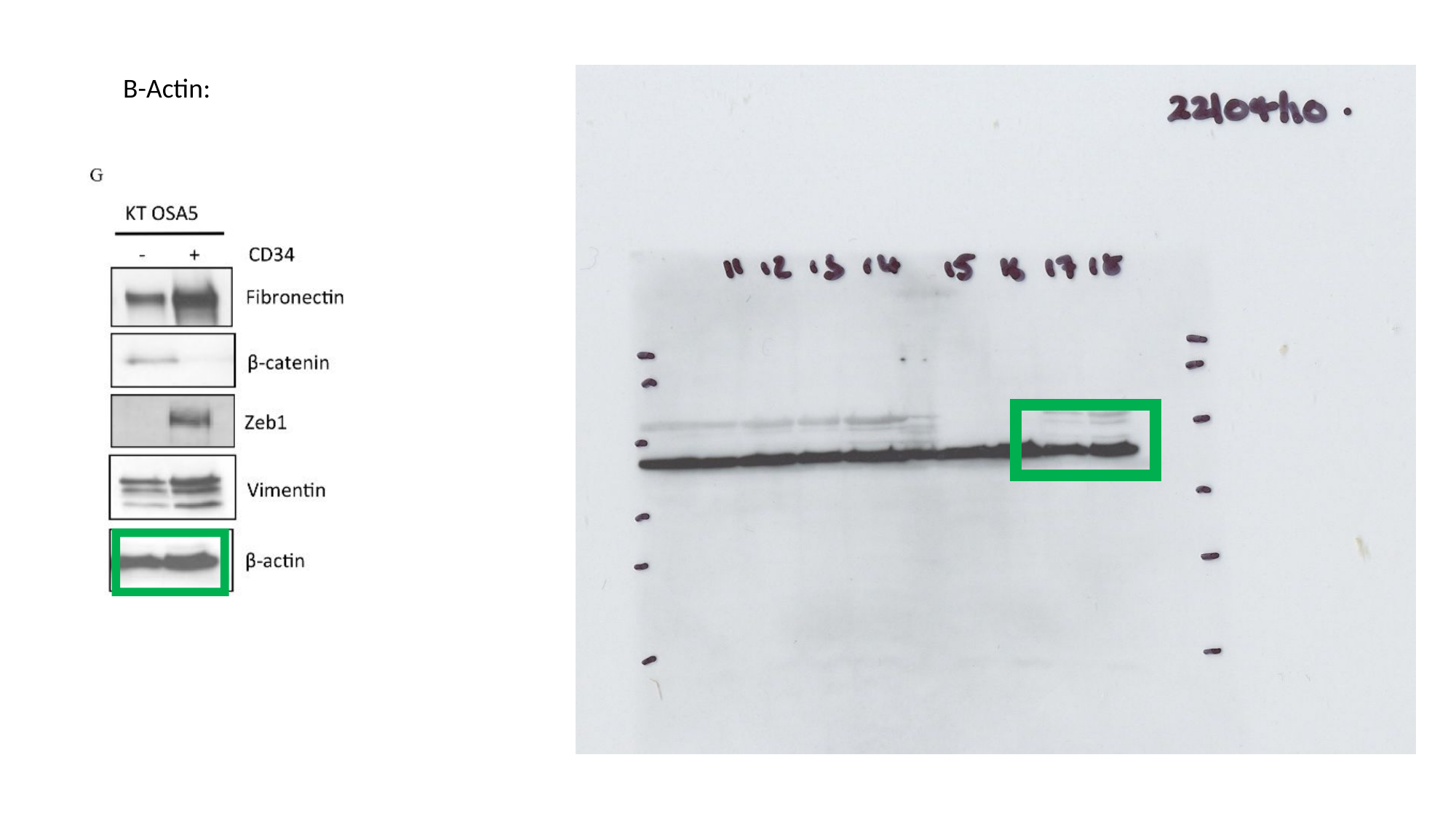

B-Actin:

Supplement: S5 File — (PPTX) [file pone.0308114.s005.pptx]
