# Supplementary material for: Editorial Note: Global Gene Expression Analysis of Canine Osteosarcoma Stem Cells Reveals a Novel Role for COX-2 in Tumour Initiation
Source: PLoS One. 2024 Aug 15;19(8):e0308114. doi: 10.1371/journal.pone.0308114 (PMC11326644; doi:10.1371/journal.pone.0308114)
Supplement: S4 File — (PPTX) [file pone.0308114.s004.pptx]

## Slide 1
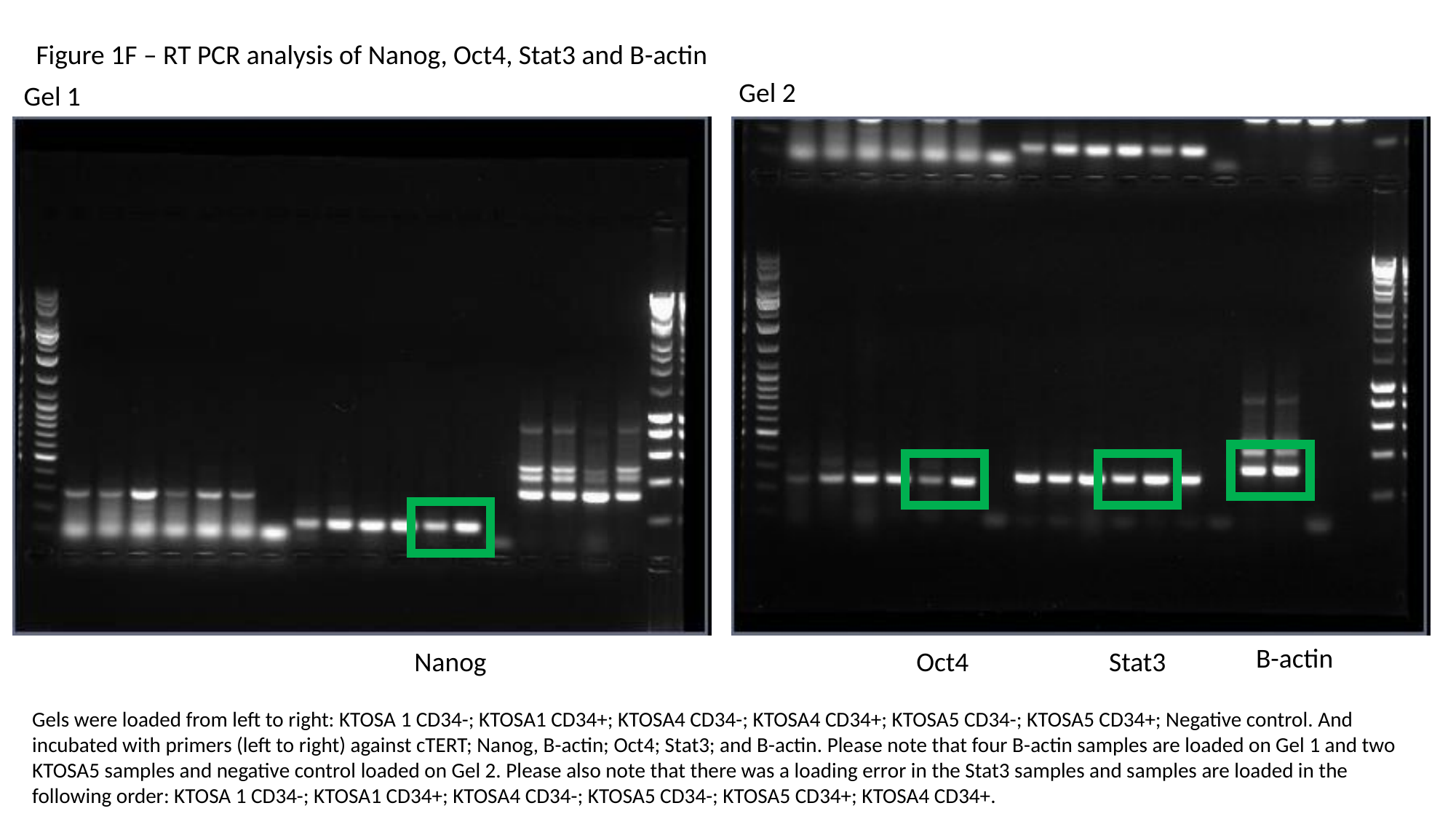

Figure 1F – RT PCR analysis of Nanog, Oct4, Stat3 and B-actin
Gel 2
Gel 1
B-actin
Nanog
Oct4
Stat3
Gels were loaded from left to right: KTOSA 1 CD34-; KTOSA1 CD34+; KTOSA4 CD34-; KTOSA4 CD34+; KTOSA5 CD34-; KTOSA5 CD34+; Negative control. And incubated with primers (left to right) against cTERT; Nanog, B-actin; Oct4; Stat3; and B-actin. Please note that four B-actin samples are loaded on Gel 1 and two KTOSA5 samples and negative control loaded on Gel 2. Please also note that there was a loading error in the Stat3 samples and samples are loaded in the following order: KTOSA 1 CD34-; KTOSA1 CD34+; KTOSA4 CD34-; KTOSA5 CD34-; KTOSA5 CD34+; KTOSA4 CD34+.
